# Papillary renal cell carcinoma with high‐ABCC2 shows an immune‐evasive profile associated with favorable response to immunotherapy

**DOI:** 10.1002/path.70001

**Published:** 2025-11-19

**Authors:** Vincent Francis Castillo, Abraam Zakhary, Fabio Rotondo, Caterina Di Ciano‐Oliveira, Malek Hamdani, Emelyn Adona, Theodorus van der Kwast, Kiril Trpkov, Rola Saleeb

**Affiliations:** ^1^ Department of Laboratory Medicine and Pathobiology University of Toronto Toronto ON Canada; ^2^ Li Ka Shing Knowledge Institute St. Michael's Hospital Toronto ON Canada; ^3^ Department of Laboratory Medicine Unity Health Toronto Toronto ON Canada; ^4^ Division of Pathology University Health Network Toronto ON Canada; ^5^ Department of Pathology and Laboratory Medicine Alberta Precision Laboratories and University of Calgary Calgary AB Canada; ^6^ Ontario Institute for Cancer Research Toronto ON Canada

**Keywords:** papillary renal cell carcinoma, kidney cancer, tumor microenvironment, ABCC2, predictive, immunotherapy, immune checkpoint inhibitor

## Abstract

The use of immune checkpoint inhibitors is a promising therapeutic strategy for metastatic papillary renal cell carcinoma (PRCC); however, predictive biomarkers remain limited. PRCCs with high ABCC2 expression represent an aggressive subset frequently associated with metastasis. The tumor microenvironment (TME) profile of these tumors remains poorly defined. This study aims to characterize the TME of PRCC in relation to its ABCC2 status. A discovery cohort of 157 ABCC2‐high PRCCs, 156 ABCC2‐low PRCCs, and 72 normal kidneys was evaluated. Using RNA sequencing data, immune cell composition, immune checkpoint markers, and immune signature scores were assessed. Validation was performed in an independent cohort (31 ABCC2‐high, 36 ABCC2‐low, and 15 normal kidneys) using RNA *in situ* hybridization (RNA‐ISH) and immunohistochemistry (IHC). ABCC2‐high PRCCs demonstrated increased infiltration of cytotoxic T cells (*p* < 0.001), M2 macrophages (*p* = 0.021), and regulatory T cells (*p* < 0.001) compared to ABCC2‐low tumors. ABCC2‐high PRCCs also had higher expression of immune checkpoint biomarkers including programmed cell death ligand 1 (PD‐L1) (*p* < 0.001). The validation cohort showed this similar TME profile. Additionally, ABCC2‐high PRCCs had higher PD‐L1 IHC positivity (combined positive score ≥ 1, *p* = 0.035; tumor proportion score ≥ 1%, *p* = 0.006) and immune predictive signature score (*p* = 0.029). NRF2–Antioxidant Response Element signaling pathway was enriched in ABCC2‐high PRCCs as evidenced by overrepresentation in pathway analysis, higher gene signature score (*p* < 0.001), and elevated transcript signals (*NFE2L2*, *p* < 0.001; *NQO1*, *p* < 0.001), compared to ABCC2‐low PRCCs. In conclusion, ABCC2‐high PRCCs are immune‐infiltrated tumors with a suppressive phenotype potentially responsive to immune checkpoint inhibitors. ABCC2 IHC may serve as a predictive biomarker to help identify patients likely to benefit from such therapy. © 2025 The Author(s). *The Journal of Pathology* published by John Wiley & Sons Ltd on behalf of The Pathological Society of Great Britain and Ireland.

## Introduction

Papillary renal cell carcinoma (PRCC) is the second most prevalent subtype of renal cell carcinoma (RCC), accounting for about 15% of cases, following clear cell RCC (CCRCC) [1]. Despite its lower incidence compared to CCRCC, metastatic PRCC is associated with a particularly poor prognosis that is often worse than metastatic CCRCC [2]. This could be partly attributed to the fact that the treatment remains challenging for locally advanced and metastatic PRCC. The treatment for PRCC has evolved, from limited options like mTOR inhibitors and tyrosine kinase inhibitors (TKIs), to a more biology‐driven therapy. For example, since the *MET* gene is overexpressed in some PRCCs, MET inhibitors, including cabozantinib and savolitinib, are currently used as first‐line therapies for metastatic PRCC lesions [3, 4, 5]. However, not all patients respond favorably to MET inhibition, and the clinical benefits have not translated into improved survival outcomes [6]. This is likely because *MET* alterations are predominantly seen in low‐grade PRCC, while a significant proportion of advanced PRCCs do not harbor *MET*‐activating mutations [7, 8, 9].

PRCC exhibits a spectrum of heterogeneity, from low‐grade or biologically indolent tumors to high‐grade or aggressive tumors [9]. The heterogeneity of PRCC is evident both morphologically, reflected in the now obsolete typing system (type 1 versus type 2), and molecularly, with more frequent *MET* overexpression and chromosome 7 and chromosome 17 gains on the indolent side versus the more common NRF2–Antioxidant Response Element (ARE) pathway activation on the aggressive side [8, 9, 10, 11]. However, many cases exhibit mixed biology and fall between these spectrum ends [10, 12, 13, 14]. This heterogeneity likely contributes to the varied suboptimal response of metastatic PRCC to current therapies. We, however, have identified a subset of aggressive PRCCs characterized by high *ABCC2* gene expression, manifested by ABCC2 immunohistochemistry (IHC) brush border expression patterns [14, 15, 16]. This group contributes significantly to the advanced PRCC cohort (supplementary material, Figure S1) [8]. Therefore, it is essential to understand the underlying biology of this PRCC group with high ABCC2 expression, to help identify possible therapeutic strategies, particularly in a metastatic setting.

Immune checkpoint inhibitors (ICIs) have transformed the treatment landscape for many solid tumors, including metastatic CCRCC [17]. ICI‐based therapies targeting the programmed cell death protein 1 (PD1)‐programmed cell death ligand 1 (PD‐L1) axis (e.g. pembroluzimab and nivolumab) are now approved as first‐line therapies for metastatic CCRCC [5, 17]. Likewise, ICIs have shown promising therapeutic potential in early‐phase PRCC clinical trials [18, 19, 20]. Recent trials focused on combining ICI with TKI or combining dual ICI agents (ipilimumab plus nivolumab) and have shown some clinical benefit [21, 22, 23]. These developments underscore the increasing potential of ICI in the treatment strategy for PRCCs.

However, a significant proportion of patients remain refractory to ICI treatments [24]. Additionally, many patients experience significant adverse effects, making it challenging to balance survival outcomes with quality‐of‐life considerations [25]. This therapeutic dilemma is further aggravated by the lack of predictive biomarkers that can identify which PRCC patients are most likely to benefit from ICI [26]. This represents a major gap in the personalization of PRCC therapy that highlights the need for further insights into the PRCC tumor microenvironment (TME).

In this study, we conducted a comprehensive evaluation of the TME of PRCC, focusing on its relation to ABCC2 status. Additionally, we sought to identify the key pathways that may account for the aggressive nature and specific immune profile of this clinically important PRCC subset.

## Materials and methods

### Ethics approval

Appropriate research ethics approvals and material transfer agreements were obtained from the corresponding institutions (Ontario Tumor Bank and Unity Health Toronto). The study cohort is outlined in the supplementary material, Figure S2.

### Initial study cohort

Fresh frozen samples of PRCC tumors (*n* = 47) and their corresponding normal kidney tissue (*n* = 42) were obtained from the Ontario Tumor Bank. Whole transcriptome sequencing was performed, and RNA sequencing (RNA‐seq) data were then normalized to transcripts per million (TPM). Additionally, RNA‐seq data from The Cancer Genome Atlas (TCGA) PRCC cohort were retrieved from the National Cancer Institute's Genomic Data Commons Data Portal (portal.gdc.cancer.gov). Outliers were excluded based on H&E morphologic evaluation, IHC profiles, and molecular data. After these exclusions, a total of 266 cases were included in the study. Furthermore, we included RNA‐seq data from normal kidney samples (*n* = 30) available in the TCGA PRCC cohort. Overall, the initial cohort consisted of 313 PRCC cases and 72 normal kidney samples.

### Validation cohort

To validate our findings from the initial cohort, we collected 67 formalin‐fixed paraffin‐embedded (FFPE) PRCC samples and 15 FFPE normal kidney samples from Unity Health Toronto. Tissue microarrays (TMA) were constructed from FFPE blocks, with three 1.2‐mm‐diameter tissue cores obtained per case. PRCC diagnosis was made using the 2022 WHO classification criteria [27] and was further confirmed using an IHC panel to exclude other RCC subtypes with papillary architecture.

### Dichotomizing cohorts into ABCC2‐high and ABCC2‐low

In the initial cohort, ABCC2 IHC was performed on 33 PRCC cases with FFPE blocks from the Ontario Tumor Bank using the ABCC2 antibody (ABCAM, Boston, MA, USA; Catalogue No.: ab187644, clone EPR10997(2), dilution 1:100) and the automated Ventana staining platform (Ventana Medical Systems, Tucson, AZ, USA) [15]. Cases were classified as ABCC2‐high (brush border on IHC, *n* = 17) or ABCC2‐low (negative/cytoplasmic on IHC, *n* = 16) by two pathologists (RS and VC). For PRCCs without FFPE blocks (*n* = 14) and for the TCGA PRCC cohort, ABCC2 status was determined by median *ABCC2* gene expression, yielding 140 in each group. In total, the initial cohort consisted of 157 ABCC2‐high PRCCs and 156 ABCC2‐low PRCCs.

For the validation cohort, PRCC cases were also stratified using ABCC2 IHC as described. After the classification, this cohort included 31 ABCC2‐high PRCCs and 36 ABCC2‐low PRCCs.

### Immune cell profiling of TME in initial PRCC cohort

To estimate the abundance of immune cell populations in the initial cohort, we utilized CIBERSORTx (https://cibersortx.stanford.edu), a machine‐learning‐based deconvolution algorithm that infers cell‐type composition from bulk RNA‐seq data [28]. Gene expression data of both PRCC tumors, and normal kidney tissues were analyzed using the LM22 gene signature matrix to determine 22 immune cell types [28].

### Immune checkpoint markers and immune signature of the initial PRCC cohort

Using the normalized RNA‐seq data, the expression levels of *PD‐L1* and other current and emerging immune checkpoint molecules [29, 30] were compared in the PRCC tumors (both high‐ABCC2 and low‐ABCC2), and in the nonneoplastic renal tissues. We also computed the JAVELIN Renal 101 immune signature score, a validated biomarker score based on the expression of 28 immune‐related genes that is used to predict tumor response to ICIs [31].

### Pathway analysis of initial PRCC cohort

Gene expression data from PRCCs with corresponding FFPE blocks (ABCC2‐high, *n* = 17; ABCC2‐low, *n* = 16) from the initial cohort were used for pathway analysis. The RNA‐seq data were processed using the DESeq2 module [32] of GenePattern (https://www.genepattern.org) [33]. We then conducted gene set enrichment analysis (GSEA) to identify overrepresented biological processes and pathways using Reactome [34] and the Hallmark gene sets from the Molecular Signatures Database [35]. A significance threshold of false discovery rate‐adjusted *p* value <0.25 was applied. In addition, the NRF2–ARE signature score [8, 36] was calculated from the RNA‐seq data of the initial PRCC cohort.

### Validation using multiplex RNA *in situ* hybridization

To confirm the findings from the initial PRCC cohort, we performed RNA *in situ* hybridization (RNA‐ISH) platform analysis on our validation cohort using the RNAscope Hiplex V2 assay (ACDBio, Newark, CA, USA, Catalogue No.: 324419) following the manufacturer's protocol. This method allows identification of up to 12 targets on the same tissue slide using probes designed to specifically hybridize to the target RNA sequences [37, 38]. We selected probes to identify specific immune cell populations including cytotoxic and regulatory T cells and M1 and M2 macrophages [38]. We also included probes targeting the NRF2–ARE‐related genes, as well as immune checkpoint genes: *PD‐L1* and *PD1*.

Quantitative image analysis was performed using the HALO *in situ* hybridization (ISH) module (Indica Labs, Albuquerque, NM, USA), which is specifically designed for the analysis of RNAscope images [39]. The percentage of immune cells was determined based on the co‐expression of immune markers (e.g. *CD3* and *CD8* for cytotoxic T cells). To quantify the expression levels of NRF2–ARE and immune checkpoint genes, we used the H‐score generated by the HALO ISH module.

### Validation using PD‐L1 IHC assay

Protein expression of *PD‐L1* was determined using PD‐L1 (22C3) IHC antibody (Agilent Dako, Santa Clara, CA, USA; clone 22C3, dilution 1:50) on the Ventana staining platform (Ventana Medical Systems). The analysis was performed on 61 PRCCs from the validation cohort, which included 25 with ABCC2‐high and 36 with ABCC2‐low. Three cores per case were evaluated by two pathologists (RS and VC), who were blinded to ABCC2 IHC status.

PD‐L1 expression was determined using the combined positive score (CPS) and tumor proportion score (TPS). A CPS of 1 and a TPS of 1% were used as cut‐offs to classify PRCC as positive for PD‐L1 expression [40].

### Statistical analyses

We used the Kruskal–Wallis test with Dunn's *post hoc* test to compare the scores from CIBERSORTx analysis and immune checkpoint gene expression among ABCC2‐high PRCC, ABCC2‐low PRCC, and nonneoplastic renal tissue groups. The same approach was applied for multiplex ISH data. PD‐L1 IHC differences were assessed using Fisher's exact test. JAVELIN Renal 101 and NRF2–ARE signature scores were compared using Mann–Whitney test. A *p* value <0.05 was considered significant. GraphPad Prism (GraphPad Software, version 9.0, San Diego, CA, USA) and SRplot (https://www.bioinformatics.com.cn) were used for statistical analyses and data visualization.

## Results

### Clinicopathological profile of study cohort

The clinicopathological characteristics of the study cohort are summarized in the Supplementary material, Tables S1 and S2.

### Immune cell profiling reveals an immunosuppressive milieu in ABCC2‐high PRCC

**Figure 1:**
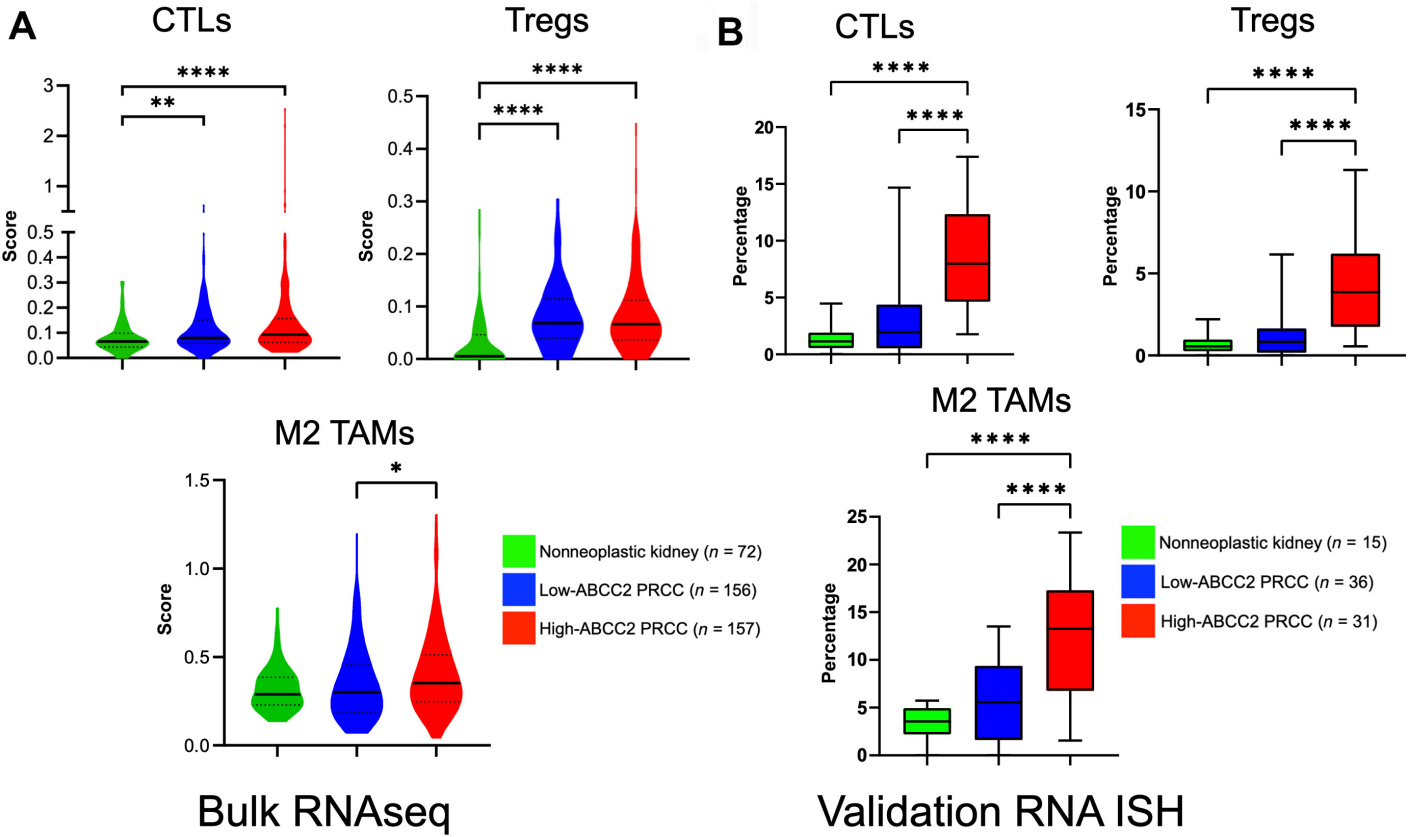
Immune transcriptomic profiling of PRCCs. CIBERSORTx analysis using (A) RNA‐seq data expressed in absolute score and (B) multiplex RNA‐ISH in terms of percentage showed higher cytotoxic T cells, regulatory T cells, and M2 tissue‐associated macrophages in ABCC2‐high PRCCs compared to ABCC2‐low PRCCs and normal kidney tissues.

We analyzed the whole transcriptomic profiles of a PRCC cohort comprising 157 ABCC2‐high and 156 ABCC2‐low, as well as 72 normal kidney samples. Using RNA‐seq data, we estimated the proportions of immune cell subsets through the CIBERSORTx deconvolution algorithm [28]. Compared to normal renal tissue, PRCCs with high‐ABCC2 expression exhibited a significant increase in cytotoxic T lymphocytes (CTL) (*p* < 0.001) and regulatory T cells (Treg) (*p* < 0.001) (Figure 1A). While the presence of CTL suggests an activated immune response, the concurrent rise in Treg indicates an immunosuppressive environment that could dampen CTL‐mediated tumor control [41, 42, 43, 44]. This immunosuppressive trend was further supported by a marked reduction in resting (*p* < 0.019) and activated dendritic cells (*p* < 0.001) in the ABCC2‐high group, compared to the normal kidney tissue. The depletion of antigen‐presenting cells, such as dendritic cells, may contribute to a dysfunctional immune response within the TME [41, 44, 45]. Other immune cell populations that were significantly lower in ABCC2‐high PRCCs, compared to normal kidney tissue, included naïve B cells (*p* < 0.001), plasma cells (*p* < 0.001), naïve CD4 T cells (*p* < 0.001), gamma delta T cells (*p* < 0.001), eosinophils (*p* < 0.001), and neutrophils (*p* = 0.038). Additionally, compared to ABCC2‐low PRCCs, ABCC2‐high PRCCs were significantly enriched with tumor‐associated macrophages (TAM), including M0 (*p* = 0.018), M1 (*p* < 0.001), and M2 (*p* = 0.021) phenotypes (Figure 1A). Notably, the increase in M2 macrophages, a phenotype associated with immunosuppression and tumor progression, suggests an immune‐evasive TME that may facilitate tumor growth [43, 46, 47].

Building on the RNA‐seq findings in our initial cohort, we proceeded to confirm our results on an independent validation cohort, which included 31 ABCC2‐high and 36 ABCC2‐low and 30 normal kidney samples, using a multiplex transcriptomic *in situ* analysis. This approach allowed visualization of immune cells through specific markers [37, 38]. Consistent with the CIBERSORTx analysis, ABCC2‐high PRCCs exhibited significantly higher levels of CTL, Treg, and M2 TAM compared to ABCC2‐low PRCCs (CTL, *p* < 0.001; Treg, *p* < 0.001; M2 TAM, *p* < 0.001) and normal kidney (CTL, *p* < 0.001; Treg, *p* < 0.001; M2 TAM, *p* < 0.001) (Figure 1B).

Overall, our findings suggest that ABCC2‐high PRCCs exhibit a tumor‐permissive milieu featuring increased CTL within a predominantly immunosuppressive microenvironment, driven by Treg and M2 TAM. This immune profile may contribute to tumor progression and immune evasion in ABCC2‐high PRCCs.

### Immunosuppressive immune checkpoint markers are overexpressed in ABCC2‐high PRCC

Since the analysis of immune cell composition in ABCC2‐high PRCC revealed an immune‐evasive landscape, we investigated the expression of immune checkpoints, which play a critical regulatory role in shaping the TME [29, 30, 43, 48].

**Figure 2:**
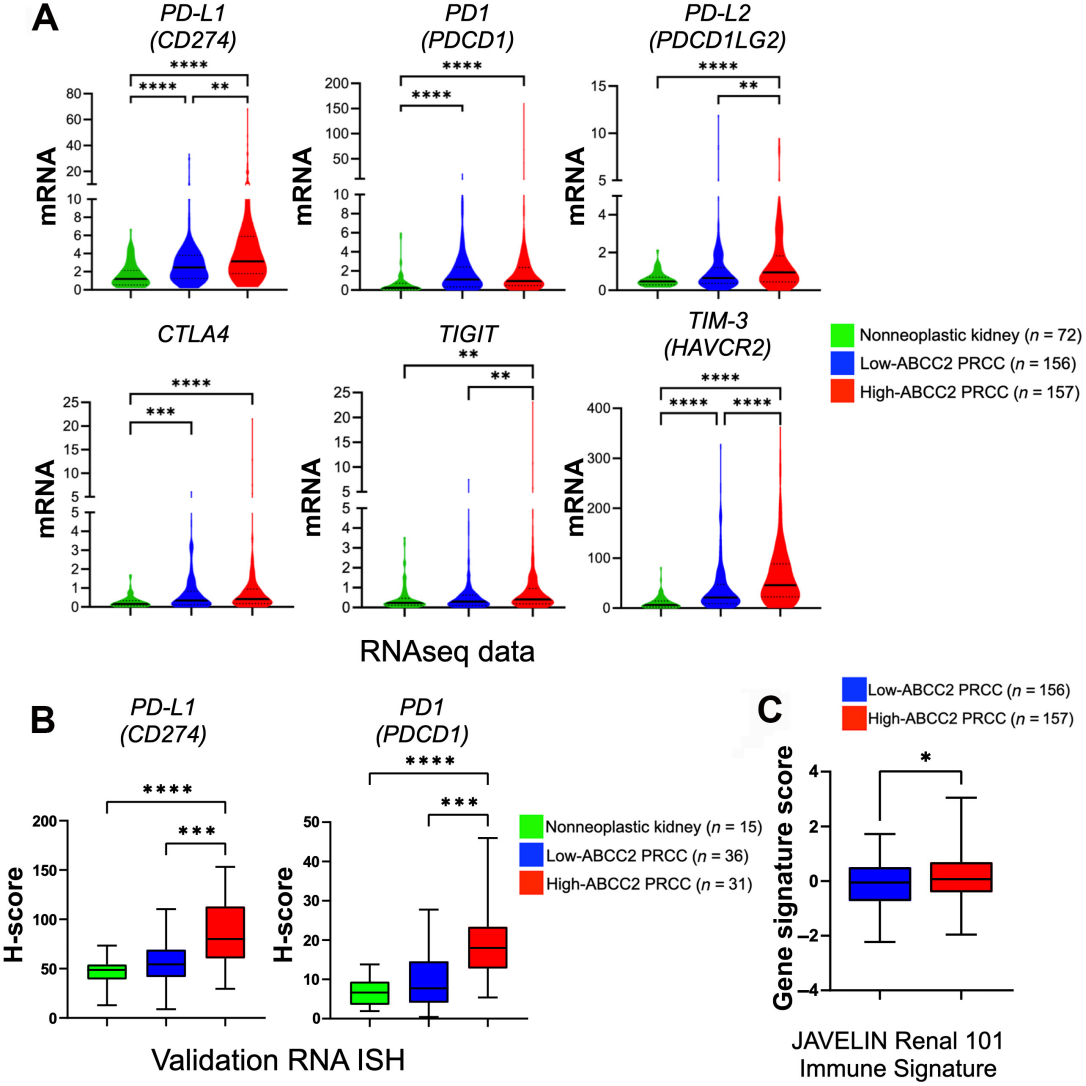
Gene expression analysis of different immune checkpoints and immune signature score in PRCC. ABCC2‐high PRCCs were associated with increased transcript levels of some immune checkpoint markers including *PD‐L1*, *PD1*, *TIGIT*, and *TIM3*, as evidenced by RNA‐seq data of (A) the initial cohort and/or (B) RNA *in situ* hybridization (RNA‐ISH) data of the validation cohort. (C) Additionally, ABCC2‐high PRCCs had higher JAVELIN Renal 101 immune score, predictive of a response to ICIs.

The gene expression data from the initial cohort showed a significant increase in *PD‐L1* gene expression in ABCC2‐high PRCCs in comparison with ABCC2‐low PRCCs (*p* = 0.001) and nonneoplastic renal tissues (*p* < 0.001) (Figure 2A). This finding was consistent with the RNA‐ISH analysis on the validation PRCC cohort, which confirmed higher *PD‐L1* expression among PRCCs with ABCC2‐high versus PRCCs with ABCC2‐low (*p* < 0.001) and normal kidney tissues (*p* < 0.001) (Figure 2B).

Although the bulk RNA‐seq data showed no significant differences in *PD1* gene expression between ABCC2‐high and ABCC2‐low PRCCs in our initial cohort, the validation RNA‐ISH assay revealed *PD1* overexpression in ABCC2‐high PRCCs (*p* = 0.001) compared to the ABCC2‐low PRCCs (Figure 2A,B). Additionally, the ABCC2‐high PRCC group exhibited significant overexpression of other immune checkpoints, such as *TIGIT* (*p* = 0.006) and *TIM*‐3 (*p* < 0.001), compared to the ABCC2‐low PRCC group in the initial cohort (Figure 2A).

**Figure 3:**
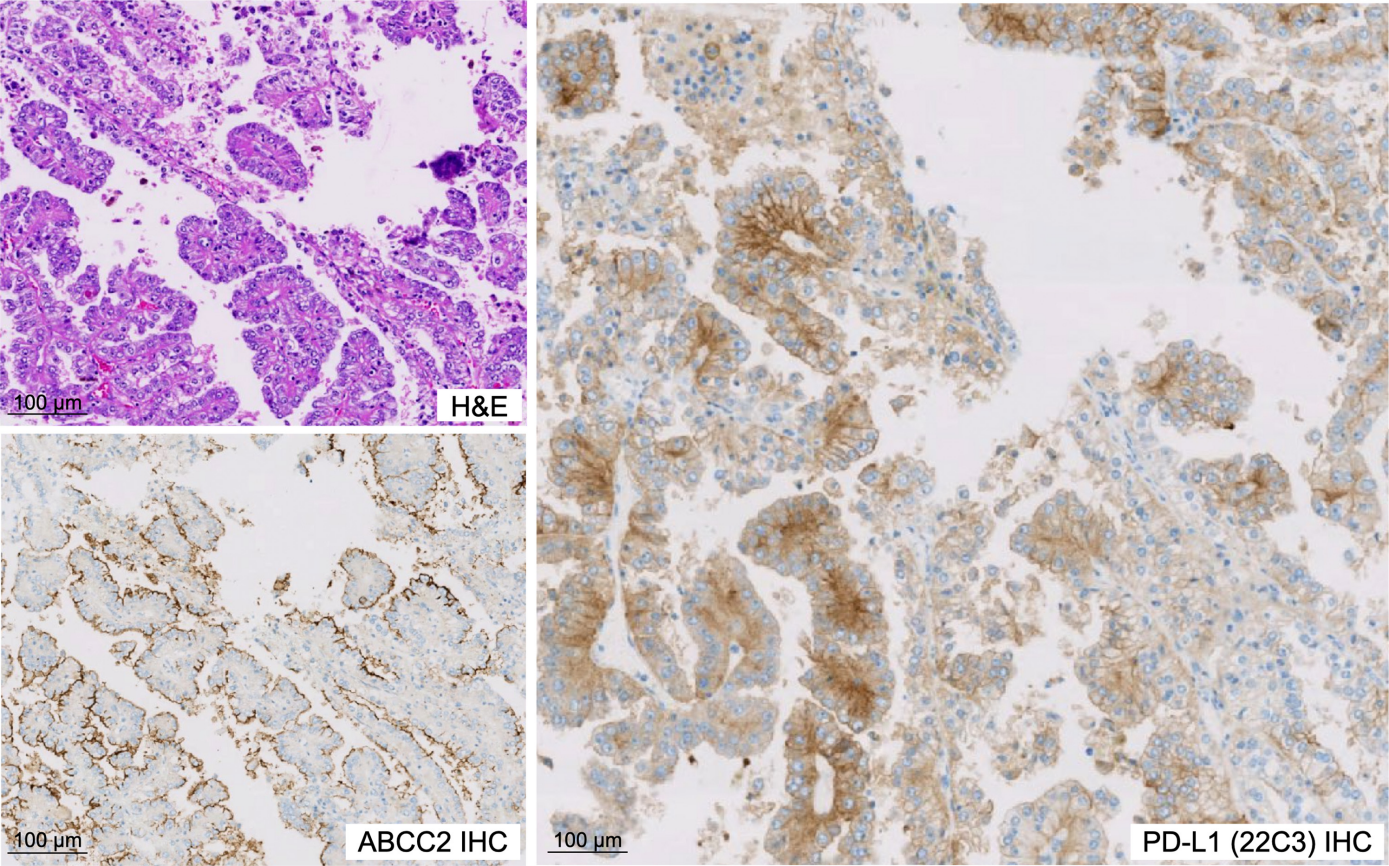
PD‐L1 IHC expression in ABCC2‐high PRCCs. Representative microscopic images showing PRCC with high ABCC2 [brush border pattern on IHC] and positive PD‐L1 IHC using both TPS ≥ 1% and CPS ≥ 1.

To further substantiate the results from our transcriptomic analyses, we assessed PD‐L1 protein expression in our validation cohort using the PD‐L1 (22C3) IHC assay. PD‐L1 positivity was observed in 19% (8/61) of PRCCs, based on a TPS ≥1% and 39% (24/61) based on a CPS ≥ 1. Among ABCC2‐high PRCCs, 28% (7/25) and 56% (14/25) were positive for PD‐L1 based on a TPS ≥ 1% and a CPS ≥ 1, respectively. Additionally, the ABCC2‐high group showed significantly higher PD‐L1 IHC expression (CPS, *p* = 0.035; TPS, *p* = 0.006) than the ABCC2‐low group (Figure 3).

Overall, ABCC2‐high PRCCs were associated with overexpression of immune checkpoints, including PD‐L1, which may facilitate evasion of immune surveillance and may promote a protumoral environment [29, 30, 43, 48], as evidenced in our immune cell profiling.

### *In‐silico* assessment shows potential response of ABCC2‐high PRCC to ICI therapy

We demonstrated that the immune landscape of ABCC2‐high PRCCs is characterized by an increase in CTL, together with higher levels of suppressive immune cells and immune checkpoints, including PD‐L1. This immune profile has been reported to correlate with a favorable response to ICI therapy in solid tumors, including RCC [44, 48]. To further investigate whether PRCCs with ABCC2‐high have a potential response to ICIs, we calculated the JAVELIN Renal 101 immune signature using the gene expression dataset from our initial cohort. High expression of this 26‐gene signature has been associated with clinical benefit in patients with CCRCC receiving ICI therapy [31]. We found that the ABCC2‐high PRCC group exhibited a higher signature score (*p* = 0.029) compared to the ABCC2‐low PRCC group (Figure 2C). This finding further supports the notion that this PRCC subset may respond favorably to ICI treatment.

### ABCC2‐high PRCC subgroups differ in immune landscape and predicted ICI responsiveness

We acknowledge that heterogeneity may exist within the ABCC2‐high PRCC subgroup. To explore this, we stratified ABCC2‐high tumors into ‘upper‐end’ and ‘lower‐end’ categories using ABCC2 IHC staining or ABCC2 RNA expression levels. Tumors in the upper‐end subgroup (≥ 50% brush border staining or above median RNA expression in that group) exhibited more immunosuppressive M2 TAM (*p* = 0.002) and higher JAVELIN immune scores (*p* = 0.032) compared to those PRCCs in the lower‐end subgroup, suggesting a potential greater responsiveness to ICIs. The complete immune profiling results are provided in the supplementary material, Table S3. These results suggest that ABCC2‐high tumors at the upper end of the spectrum may be more immunosuppressive, driven by higher M2 macrophage abundance and potentially more responsive to ICI therapy compared to lower‐end ABCC2‐high tumors.

### Transcriptomic analysis shows enrichment of NRF2–ARE signaling pathway in ABCC2‐high PRCCs

To explore the biologic mechanisms underlying the aggressive nature and immune‐evasive profile of ABCC2‐high PRCCs, we performed a pathway analysis using transcriptomic data of 33 PRCCs (17 ABCC2‐high, 16 ABCC2‐low) from our initial cohort. The ABCC2 status of these cases was determined by ABCC2 IHC, which was previously validated as a prognostic marker in PRCC [15, 16]. Relative to ABCC2‐low PRCCs, ABCC2‐high PRCCs were significantly enriched with 357 Reactome pathways and 29 Hallmark gene sets (as shown in the supplementary material, Figure S3, depicting the differentially expressed genes and top Reactome and Hallmark pathways overrepresented in ABCC2‐high PRCCs).

**Figure 4:**
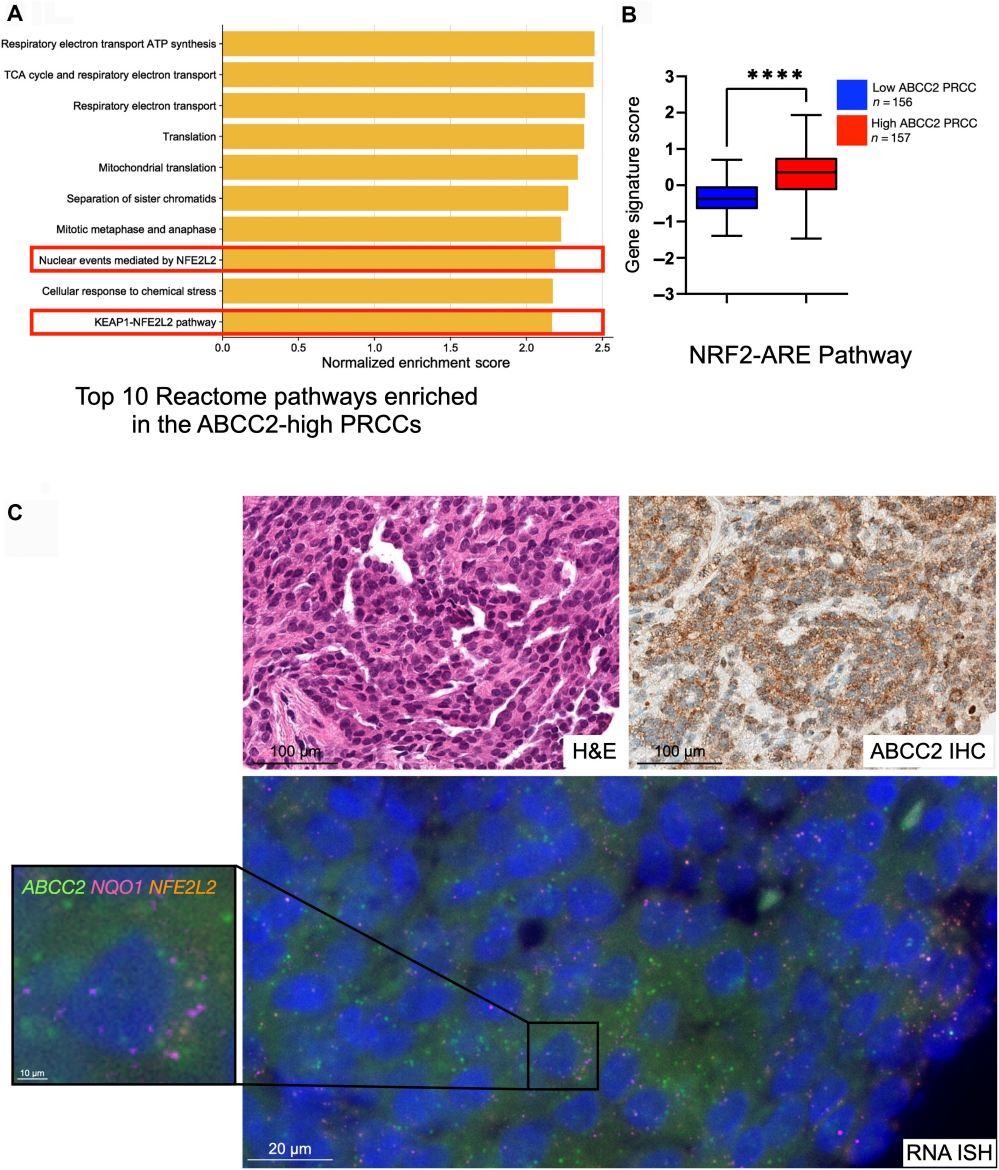
Enrichment of NRF2–ARE pathway in ABCC‐high PRCCs. (A) NRF2–ARE signaling pathways (highlighted) were among the top enriched pathways in ABCC2‐high PRCCs. (B) The NRF2–ARE signature score was significantly higher in ABCC2‐high PRCCs based on the initial cohort dataset. (C) Validation using multiplex RNA‐ISH confirmed increased NRF2–ARE‐related transcripts, including in *NQO1* (pink) and *NFE2L2* (orange), in this PRCC subset.

Among the enriched pathways, the NRF2–ARE signaling cascade was one of the most prominent (Figure 4A). This pathway was previously reported to be enriched in aggressive PRCCs in the TCGA cohort [8, 13] and has been linked to immune‐evasive/exhaustive profile in other solid tumors [49, 50, 51]. Given these associations, we prioritized the validation of NRF2–ARE activity in ABCC2‐high PRCCs. First, we calculated the NRF2–ARE pathway signature using RNA‐seq data from our initial cohort. Indeed, PRCCs expressing high ABCC2 showed a significantly higher NRF2–ARE signature score (*p* < 0.001) compared to PRCC with low ABCC2 expression (Figure 4B). Next, we examined the spatial expression of NRF2–ARE‐related genes, *NFE2L2* and *NQO1*, using an RNA‐ISH assay in the validation cohort. Our *in situ* analysis further confirmed this finding, showing that biomarkers specific to the NRF2–ARE pathway were significantly overexpressed in the ABCC2‐high group compared to the ABCC2‐low group (*NFE2L2*, *p* < 0.001; *NQO1, p* < 0.001) and the normal kidney tissue group (*NFE2L2*, *p* = 0.014; *NQO1*, *p* < 0.001) (Figure 4C). These findings suggest that the NRF2–ARE pathway may play a role in promoting aggressive tumor behavior and immune evasion in ABCC2‐high PRCCs.

## Discussion

In this study, we focused on PRCCs characterized by high‐ABCC2 expression, which can be clinically detected by its brush border reactivity pattern on ABCC2 IHC. This subset is associated with worse clinical outcome, including a higher likelihood of advanced and metastatic disease [9]. In view of the increasing favorable evidence of ICI‐based therapies, used either alone or in combination, for managing metastatic PRCCs [18, 19, 20, 21, 22, 23], we sought to explore whether patients with PRCCs showing high ABCC2 expression exhibit immune characteristics that make them amenable to such immunotherapy. Our comprehensive immune profiling of PRCCs, incorporating RNA‐seq, RNA‐ISH, and IHC data in relation to their ABCC2 expression status revealed an immune‐evasive milieu in ABCC2‐high PRCCs, suggesting that this subset may have an increased likelihood of responding to ICI‐based therapy.

Based on our immune transcriptomic profiling, we demonstrated that ABCC2‐high PRCCs were infiltrated by CTL; however, the higher proportions of immune inhibitory cells including Treg and M2 TAM could impair the functions of CTL and could promote tumor progression [46, 47, 48, 52]. In RCC, increased infiltration of Treg and M2 TAM has been linked to poor prognosis [52]. The COSMIC‐313 trial update recently reported that RCC patients with adverse clinical outcomes had higher levels of M2 TAM [53]. Notably, M2 TAM and Treg work synergistically to suppress the antitumor immunity [52]. M2 TAM recruit Treg into the TME, where Treg further suppress CTL, both directly and indirectly, while also promoting TAM polarization to M2 phenotype [44, 48, 54]. Additionally, we observed a reduction in activated dendritic cells in ABCC2‐high PRCCs, impairing antigen presentation and reducing tumor recognition. This may be attributed to Treg‐mediated suppression of dendritic cells [44].

To further characterize the TME of ABCC2‐high PRCCs, we examined the expression of some of the main immunosuppressive checkpoint ligands and receptors. Transcriptomic analysis revealed a significant overexpression of *PD‐1* and *PD‐L1* in ABCC2‐high PRCCs. To validate this finding at the protein level, we performed IHC using the PD‐L1 (22C3) antibody, and we found that high‐ABCC2 PRCCs were associated with higher PD‐L1 positivity compared to low‐ABCC2 PRCCs, using TPS ≥1% and CPS ≥1 as cut‐offs.

Clinically, PD‐L1 expression has been linked to prognosis and immunotherapy response in PRCCs. In a previous study by Naffrichoux *et al*, 28% (19/68) of metastatic PRCC cases were PD‐L1 positive (using CPS ≥1 cut‐off), and these PD‐L1‐positive PRCCs were associated with shorter overall survival [55]. Similarly, the KEYNOTE‐427 trial reported that non‐CCRCCs with high PD‐L1 expression, as measured by CPS ≥ 1, demonstrated a higher objective response rate to pembrolizumab [20]. In the recent SUNNIFORECAST trial, patients with non‐CCRCCs who had a PD‐L1 CPS ≥ 1 showed clinical benefit from treatment with a combination of ipilimumab and nivolumab [23].

Of note, the predictive value of PD‐L1 IHC in PRCC remains limited and inconsistent [56, 57, 58]. ABCC2 IHC may serve as a valuable complementary biomarker, as ABCC2‐high PRCCs are associated not only with higher PD‐L1 expression but also with increased expression of other ICI‐relevant markers and a greater immune‐predictive signature score, as discussed below. This could help identify additional potential ICI responders beyond what PD‐L1 IHC alone may capture. We also found an overexpression of other immune checkpoint genes, such as *TIGIT* and *TIM3*, in PRCCs with high ABCC2 expression. These emerging immune checkpoints for which the ABCC2‐high PRCC group could potentially benefit from are currently being evaluated in clinical trials [29, 30].

The association between elevated immune checkpoint markers and an immune‐evasive TME has been observed, and studies have shown that CTL exhibiting high levels of immune checkpoint or ‘exhausted’ markers are often associated with increased infiltration of M2 TAM and Treg [46, 47]. In ABCC2‐high PRCCs, CTL exhibited an ‘exhausted’ phenotype [59, 60], as evidenced by the significant overexpression of immune checkpoints, including PD‐L1, within the TME. Reversing this T‐cell exhaustion through ICI therapy, thereby reactivating antitumor immunity, could serve as an effective therapeutic strategy for this tumor type, as demonstrated in other cancers [59, 60, 61, 62].

Additionally, we assessed the potential responsiveness of ABCC2‐high PRCCs to ICI therapy by computing for JAVELIN Renal 101 immune signature score. This score was associated with improved progression‐free survival in patients receiving ICI therapy in the JAVELIN Renal 101 trial [31]. This signature score was indeed significantly higher in the PRCCs with increased ABCC2, suggesting that this subset may benefit from ICI treatment [31]. We applied this immune signature as a surrogate marker in the absence of clinical ICI response data in our study cohort, recognizing that it was developed and validated in CCRCCs. While this approach is not definitive for PRCCs, our findings suggest that ABCC2‐high tumors may harbor features linked to ICI responsiveness. These results are exploratory, and further validation of ABCC2 as a predictive biomarker in PRCC is warranted, either through a retrospective analysis of clinical trial data or by including ABCC2 status into the design of future ICI‐based clinical trials.

Given the high immune signature score in ABCC2‐high PRCCs, the modest PD‐L1 positivity (28% by TPS, 56% by CPS) suggests that additional immunoregulatory pathways like *TIGIT* and *TIM3* may be involved. This highlights the need to consider a broader immune checkpoint profile beyond PD‐L1 alone to better understand the immune‐evasive microenvironment of ABCC2‐high tumors.

Within the ABCC2‐high PRCC subgroup, we observed heterogeneity, where tumors at the upper end of ABCC2 expression showed greater enrichment of immunosuppressive M2 TAM and higher JAVELIN immune scores compared to those at the lower end. Further evaluation of this ABCC2‐high ‘upper end’ subset, with larger sample cohorts to validate our results is warranted.

Although the TME of PRCCs has not been fully characterized, a recent study by de Vries‐Brilland *et al* [63] conducted a comprehensive evaluation of the TME in PRCC patients and identified a distinct subset through immune‐related transcriptomic expression analysis. This cluster was characterized by a higher proportion of CTL, B cells, and NK cells, together with increased expression of immune checkpoint markers such as PD‐L1, PD1, TIGIT, LAG3, and CTLA‐4. It also exhibited a higher predictive signature score for responsiveness to ICI therapy, including high JAVELIN Renal 101 immune score. The therapeutic relevance of this immune group was further supported by findings from *post hoc* analysis of the AXIPAP trial cohort, which demonstrated a higher objective response rate to immunotherapy in this subset. Given the similarities in terms of the observed immune profile, PRCC tumors with high ABCC2 expression may belong to this immune‐responsive cluster.

Combining *MET* inhibitors with ICI is a promising strategy under investigation in several clinical trials, with encouraging results [21, 22]. Anti‐angiogenic TKIs can normalize tumor vasculature, enhance T effector cell infiltration, and reduce immunosuppressive myeloid cells, while MET inhibition may further modulate antitumor immunity via VEGF‐axis regulation and macrophage activation [58]. As such, ABCC2‐high PRCCs may particularly benefit from this combination therapy.

We also demonstrated an association between ABCC2‐high PRCCs and the NRF2–ARE pathway. We found an overrepresentation of NRF2–ARE in our pathway analysis and a significantly higher NRF2–ARE signature score in PRCCs with high ABCC2 compared to those with low ABCC2. Additionally, multiplex ISH analysis revealed that NRF2–ARE biomarkers, *NFE2L2* and *NQO1*, were significantly elevated in PRCCs with high ABCC2 expression. The NRF2–ARE pathway was previously identified in aggressive PRCCs [8, 13]. This pathway functions as a key cellular defense mechanism against exogenous and endogenous stressors by enhancing the transcription of cytoprotective genes, such as *NQO1*, and multidrug transporters like ABCC2 [64, 65, 66]. Tumor cells exploit this pathway through continuous and uncontrolled hyperactivation, which promotes their aggressiveness and resistance to chemotherapeutic agents and causes evasion of natural immune responses [64, 65, 66]. For example, activation of NRF2 in cancer cells can shift the macrophage population toward an M2‐like state, which inhibits the antitumor inflammation and facilitates tumor progression [67]. Notably, the NRF2–ARE gene signature was also significantly overexpressed in the immune cluster identified by de Vries‐Brilland *et al* [63], further suggesting that ABCC2‐high PRCCs are included within their immune‐clustered PRCC cases. However, the potential role of the NRF2–ARE pathway in creating an immune‐suppressive environment in PRCCs is still relatively unexplored. Co‐localization studies (e.g. NRF2+ tumor cells adjacent to M2 macrophages), as well as mechanistic investigations, would help strengthen this association and clarify the role of NRF2–ARE pathway in this context.

Other studies have also supported the association between NRF2–ARE signaling and the PD‐L1 expression in various solid tumors, including non‐small cell lung carcinoma, hepatocellular carcinoma, colorectal carcinoma, and melanoma [49, 50, 51, 68]. This connection suggests a plausible explanation for the observed relationship between ABCC2 and PD‐L1, as NRF2 acts as an upstream regulator of both ABCC2 and PD‐L1. Alternatively, it is also possible that ABCC2 induces PD‐L1 expression through a mechanism independent of the NRF2–ARE pathway. ABC transporters, including ABCC2, have been reported to have a role in regulating cellular functions of immune cells in the TME through active transport of cytokines and other inflammatory mediators [69, 70, 71]. In particular, ABCC2 itself plays an active role in the transport of LTC4, which can influence the composition and activity of infiltrating immune cells within the TME. LTC4 promotes chemotaxis and activation of eosinophils, mast cells, macrophages, and dendritic cells [72, 73, 74]. The potential role of ABCC2 in modulating the TME warrants further investigation in future mechanistic studies.

While NRF2–ARE pathway activation is upstream of ABCC2, we selected ABCC2 as our biomarker because ABCC2 was the only biomarker in the NRF2–ARE pathway consistently and reproducibly associated with survival outcomes across multiple cohorts [13, 14, 15, 16]. In addition, IHC‐based detection of ABCC2 is already clinically validated in PRCCs [15, 16], whereas standardized assessments of NRF2–ARE signature activity have not been established.

A limitation to our study is that the complex interplay of immune cells within the TME, as evidenced by the coexistence of increased CTL with Treg and M2 TAM, may not be fully explained by our single‐time‐point analysis. Assessment of functional and mechanistic studies on exhaustion markers in immune cells, including CTL and TAM, and a detailed characterization of subsets like naïve and memory CTL would be beneficial future steps to validate the finding generated by our tumor and TME profiling analysis.

In conclusion, ABCC2‐high PRCCs are characterized by an immune‐evasive microenvironment, potentially driven by activation of the NRF2–ARE signaling pathway and the direct influence of ABCC2 on the TME. These findings provide a strong rationale for considering ICI therapy as a promising treatment option for this aggressive subset, which can be reliably identified through ABCC2 IHC. Our findings provide compelling evidence for further experimental and prospective studies to validate the therapeutic potential of ICI therapy in PRCCs with high ABCC2 expression.

## Author contributions statement

RS and VFC conceptualized and designed the study. VFC performed data analysis, interpretation and statistical analysis. VFC wrote the manuscript draft. VFC, KT, TvdK and RS contributed to manuscript review and revision. AZ, FR, CDC‐O, MH and EA provided technical and material support. All authors listed read and approved of the final paper.

## Supporting information


**Supplementary materials** **and methods**
**Figure S1.** ABCC2 expression in advanced PRCCs in TCGA cohort
**Figure S2.** Study cohort
**Figure S3.** Differentially expressed genes and overrepresented pathways in ABCC2‐high PRCCs
**Table S1.** Clinicopathological characteristics of discovery cohort
**Table S2.** Clinicopathological characteristics of validation cohort
**Table S3.** Immune profiling result of ABCC2‐high PRCCs divided into ‘upper‐end’ and ‘lower‐end’

## Data Availability

The data that support the findings of this study are available from the corresponding author upon reasonable request.
