# Supplementary material for: Papillary renal cell carcinoma with high‐ABCC2 shows an immune‐evasive profile associated with favorable response to immunotherapy
Source: J Pathol. 2025 Nov 19;268(2):188–99. doi: 10.1002/path.70001 (PMC12805615; doi:10.1002/path.70001)
Supplement: Supplementary file 1 — Supplementary materials and methods Figure S1. ABCC2 expression in advanced PRCCs in TCGA cohort Figure S2. Study cohort Figure S3. Differentially expressed genes and overrepresented pathways in ABCC2‐high PRCCs Table S1. Clinicopathological characteristics of discovery cohort Table S2. Clinicopathological characteristics of validation cohort Table S3. Immune profiling result of ABCC2‐high PRCCs divided into ‘upper‐end’ and ‘lower‐end’ [file PATH-268-188-s001.docx]

**Papillary renal cell carcinoma with high ABCC2 shows an immune-evasive profile associated with favorable response to immunotherapy**

VF Castillo Burguet *et al. J Pathol* <https://doi.org/10.1002/path.70001>

**Supplementary materials and methods**

**Supplementary Figures S1–S3**

**Supplementary Tables S1–S3**

**Supplementary materials and methods**

**Concordance between ABCC2 IHC and ABCC2 RNA-seq**

We assessed the concordance between ABCC2 protein expression by IHC and RNA-seq expression level in a subset of our study cohort. Specifically, we examined 33 samples with corresponding ABCC2 IHC and RNA expression data. Among the 18 samples classified as ABCC2-high by IHC (brush border staining), 94% (17/18) exhibited transcript levels above the median cut-off. Conversely, among the 16 samples classified as ABCC2-low by IHC (negative staining), 94% (15/16) had transcript levels below the median cutoff. This high concordance supports the use of the median transcript expression value as a surrogate for ABCC2 status in cases lacking IHC data, consistent with our previous studies [15,16], which reported approximately equal proportions of ABCC2-high and ABCC2-low PRCC cases by IHC.

**Immune cell profiling on TME of initial PRCC cohort**

To estimate the quantity of immune cell population in the initial cohort, we utilized CIBERSORTx (<https://cibersortx.stanford.edu>), a machine-learning-based deconvolution algorithm that infers cell-type composition from bulk RNA-seq data [28]. Gene expression data (in TPM) of both PRCC tumors and normal kidney tissues were uploaded to the CIBERSORTx web portal and analyzed using the LM22 gene signature matrix, which comprises 547 genes to determine 22 immune cell types. These include T cell types [CD8+, CD4+ naïve, CD4+ memory resting, CD4+ memory activated, follicular helper, regulatory (Treg), gamma delta], B cells (naïve, memory, plasma), NK cells (resting, activated), monocytes, macrophages (M0, M1, M2), dendritic cells (resting, activated), mast cells (resting, activated), eosinophils, and neutrophils [28].

The analysis was performed using 1,000 permutations in absolute mode to generate an absolute score for each immune cell type in each sample, enabling direct comparison of immune infiltration across PRCC subgroups and normal kidney tissues. Only results with a *p* value <0.05 were considered for further analysis.

**Immune checkpoint markers and immune signature of initial PRCC cohort**

Using the normalized RNA-seq data (TPM), gene expression of *PD-L1* and other current and emerging immune checkpoint expression, including *PD1*, *PD-L2*, *CTLA-4*, *LAG-3*, *TIGIT*, *TIM-3*, *VISTA*, *BTLA*, and *B7-H3* [29,30], were compared in the PRCC tumors (both high ABCC2 and low ABCC2) and in the nonneoplastic renal tissues.

We also computed the JAVELIN Renal 101 immune signature score, a validated biomarker score based on the expression of 28 immune-related genes (*CD3G*, *CD3E*, *CD8B*, *THEMIS*, *TRAT1*, *GRAP2*, *CD247*, *CD2*, *CD96*, *PRF1*, *CD6*, *IL7R*, *ITK*, *GPR18*, *EOMES*, *SIT1*, *NLRC3*, *CD244*, *KLRD1*, *SH2D1A*, *CCL5*, *XCL2*, *CST7*, *GFI1*, *KCNA3*, and *PSTPIP1*) [31]. Originally developed in CCRCCs, this signature score is used to predict tumor response to ICI [31]. The transcript levels (in TPM) of each immune-related gene were obtained from the RNA sequencing data, and the z-score for each gene was computed. The immune signature score was then determined by calculating the median of the z-scores of all genes in each case.

**Pathway analysis of initial PRCC cohort**

Gene expression data from PRCCs with corresponding FFPE blocks (ABCC2-high, *n* = 17; ABCC2-low, *n* = 16) of the initial cohort were used for pathway analysis. Raw count data from RNA-seq were processed using the DESeq2 module [32] of GenePattern (<https://www.genepattern.org>) [33]. The data were normalized for library size differences, allowing for the identification of differentially expressed genes between ABCC2-high and ABCC2-low groups. For each gene, a rank value was assigned based on the log2 fold-change (log2FC) and adjusted *p* values. We then conducted a GSEA on the ranked list of genes to identify overrepresented biological processes and pathways using Reactome [34] and Hallmark gene sets from the Molecular Signatures Database [35]. A significance threshold of false discovery rate-adjusted *p* value < 0.25 was applied.

In addition, NRF2-ARE signature score, which includes the genes *ABCC2*, *ACTA1*, *ACTA2*, *ACTG2*, *EPHX1*, *FTL*, *GCLM*, *GPX2*, *GSR*, *GSTA1*, *GSTA2*, *NQO1*, *PRKCE*, *SQSTM1*, and *TXNRD1* [8,36], was calculated from the RNA-seq data (TPM) of the initial PRCC cohort. The score was computed by taking the median of the z-scores of the NRF-ARE genes in each sample.

**Validation using multiplex RNA *in situ* hybridization**

To confirm the findings from the initial PRCC cohort, we performed an RNA-ISH platform analysis using the RNAscope Hiplex V2 assay (ACDBio, Newark, CA, USA, Catalogue No.: 324419) on our validation cohort. We selected the following probes to identify specific immune cell populations: *CD3* and *CD8* for cytotoxic T cells, *CD3*, *CD8*, and *FOXP3* for regulatory T cells, *CD68* for M1 macrophages, and *CD68*, *CD163*, and *ARG1* for M2 macrophages [38]. In addition, we included probes targeting the NRF2-ARE related genes *ABCC2*, *NFE2L2*, and *NQO1*, as well as immune checkpoint genes *PD-L1* and *PD1*.

In brief, four cycles of label probe hybridization and scanning were performed to detect all target probes [37]. All four scanned images were then merged into a single file using the HALO image registration module, followed by quantitative image analysis using HALO ISH module designed for RNAscope image (Indica Labs, Albuquerque, NM, USA) [39].

The percentage of immune cells was determined based on the co-expression of immune markers (e.g. *CD3* and *CD8* for cytotoxic T cells) within the tumor parenchyma. To quantify the expression levels of NRF2-ARE and immune checkpoint genes, we used the H-score generated by the HALO ISH module. The H-score is determined by categorizing the cells into one of four bins based on the number of transcript signals or dots per cell. The H-score is then calculated using the following formula: Σ (bin number × percentage of cells per bin) [39].


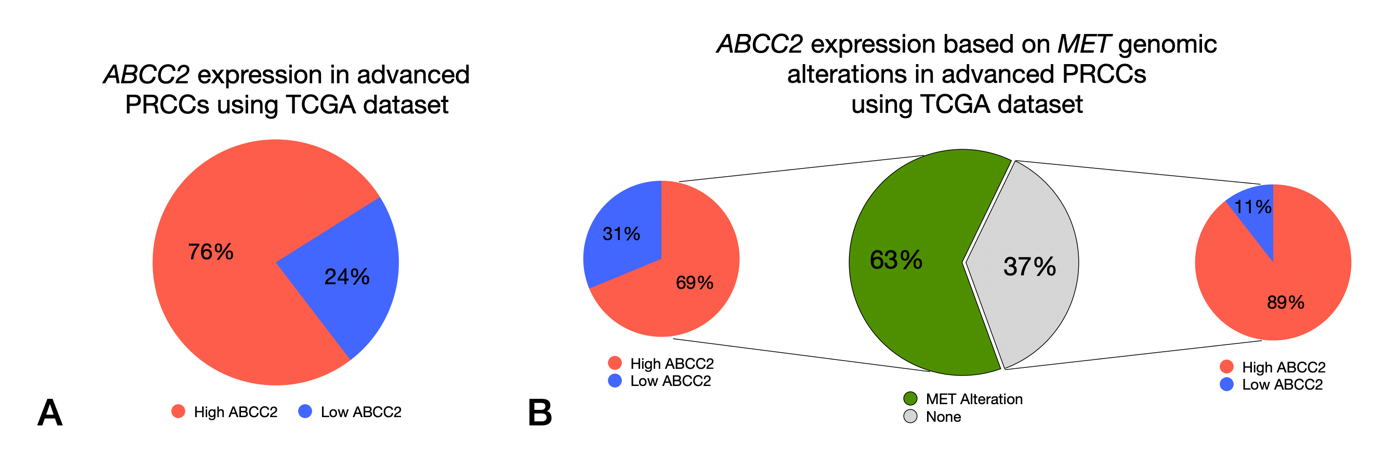


**Figure S1. ABCC2 expression in advanced PRCCs in TCGA cohort.** (A) A high proportion (76%) of advanced PRCCs exhibited high ABCC2 expression. (B) 37% of advanced PRCCs had no alteration in MET gene, and among these, the majority (89%) were ABCC2-high PRCCs. PRCCs were classified into high and low ABCC2 based on the median cutoff of *ABCC2* gene expression.


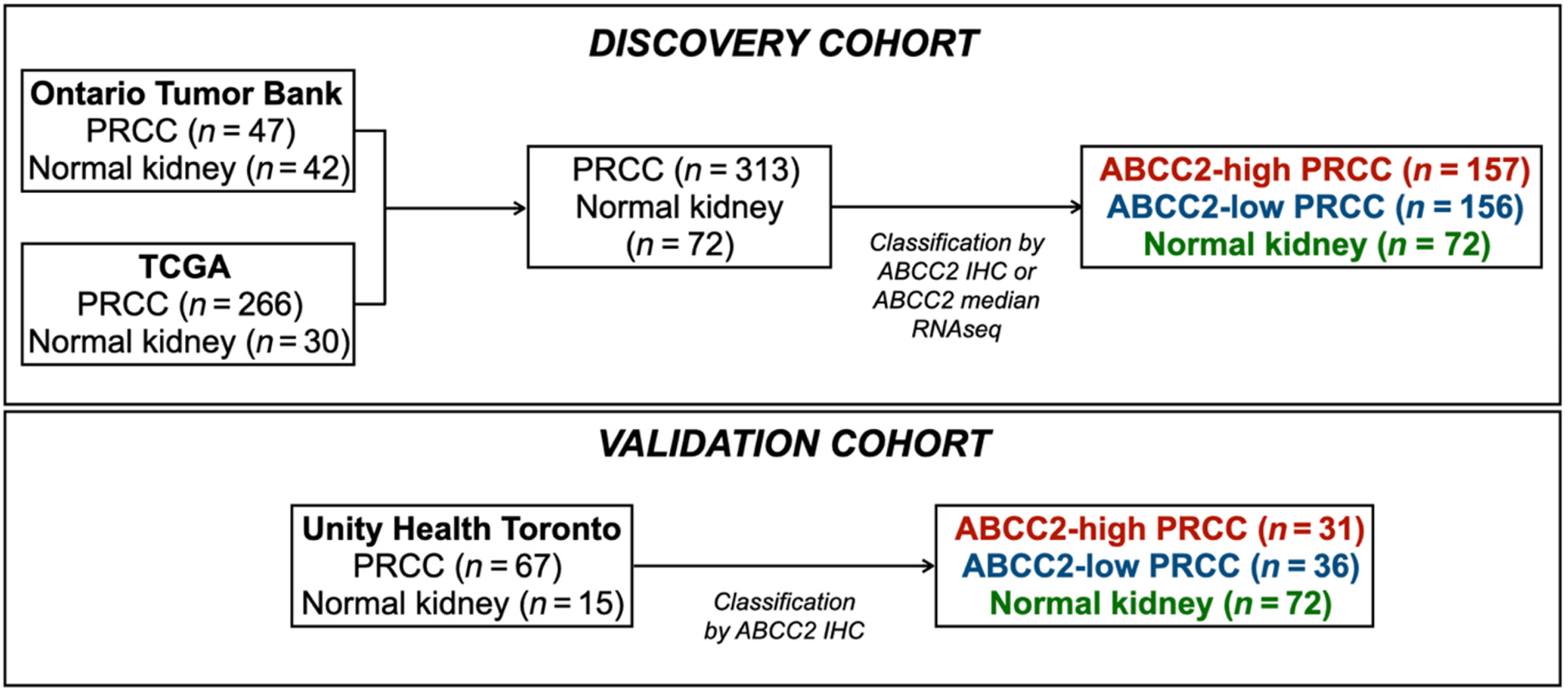


**Figure S2. Study cohort.** PRCC, papillary renal cell carcinoma; IHC, immunohistochemistry.


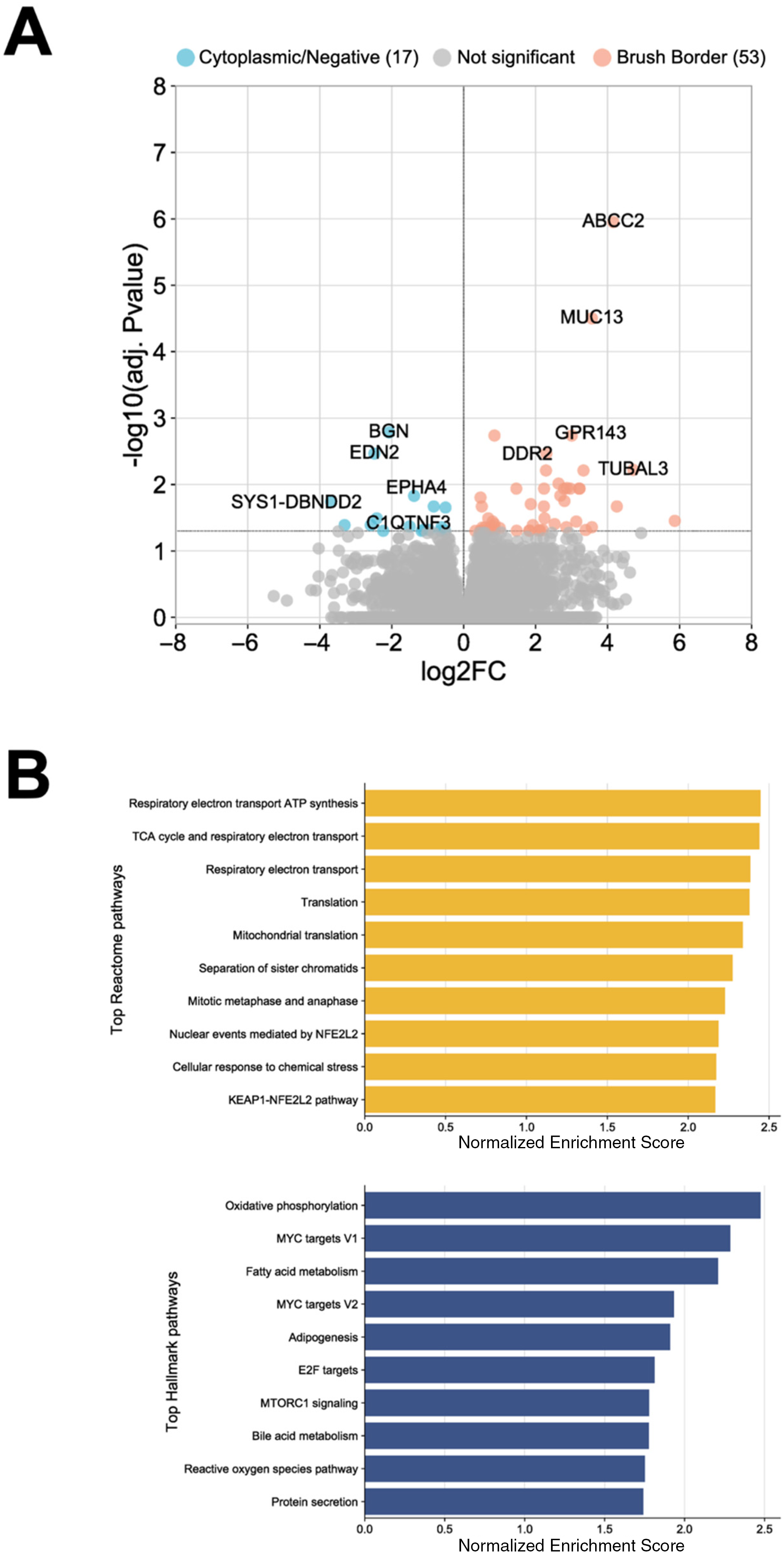


**Figure S3.** **Differentially expressed genes and overrepresented pathways in ABCC2-high PRCCs.** (A) Volcano plot showing highly expressed genes in brush border or ABCC2-high group (red dots) and negative/cytoplasmic or ABCC2-low group (blue dots). Top 10 upregulated genes for each group were annotated. (B) Top 10 Reactome and Hallmark pathways enriched in PRCC with high ABCC2.

| **Clinicopathological parameters** | **ABCC2-low PRCC**  **(*n* = 156)** | **ABCC2-high PRCC**  **(*n* = 157)** |
| --- | --- | --- |
| Age (mean, SD, years) | 61 ± 11 | 64 ± 12 |
| Sex, *n* (%) |  |  |
| Male | 123 (79) | 117 (75) |
| Female | 33 (21) | 40 (25) |
| Tumor size (mean, SD, cm)* | 5.5 ± 3.1 | 5.6 ± 3.1 |
| WHO/ISUP grade, *n* (%)* |  |  |
| 1 | 1 (4) | 0 (0) |
| 2 | 14 (59) | 10 (21) |
| 3 | 8 (33) | 12 (25) |
| 4 | 1 (4) | 2 (4) |
| Stage, *n* (%)** |  |  |
| I | 123 (79) | 90 (57) |
| II | 18 (12) | 16 (10) |
| III | 11 (7) | 44 (28) |
| IV | 3 (2) | 6 (4) |
| Distant metastasis, *n* (%) | 2 (1) | 6 (4) |
| **SD = standard deviation**  ***** Tumor size and WHO/ISUP grade data were not available for cases in TCGA cohort.  ****** Staging information was missing for two cases in TCGA cohort. | | |

**Table S1.** Clinicopathological characteristics of initial PRCC cohort (OICR and TCGA).

PRCC, papillary renal cell carcinoma; OICR, Ontario Institute for Cancer Research; TCGA, The Cancer Genome Atlas; WHO/ISUP, World Health Organization/International Society of Urological Pathology.

**Table S2.** Clinicopathological characteristics of validation PRCC cohort.

| **Clinicopathological parameters** | **ABCC2-low PRCC**  **(*n* = 36)** | **ABCC2-high PRCC**  **(*n* = 31)** |
| --- | --- | --- |
| Age (mean, SD, years) | 60 ± 12 | 62 ± 11 |
| Sex, *n* (%) |  | |
| Male | 29 (81) | 27 (87) |
| Female | 7 (19) | 4 (13) |
| Tumor size (mean, SD, cm) | 4.3 ± 3.2 | 4.5 ± 3.1 |
| WHO/ISUP grade, *n* (%) |  | |
| 1 | 3 (8) | 0 (0) |
| 2 | 23 (64) | 10 (32) |
| 3 | 10 (28) | 21 (68) |
| 4 | 0 (0) | 0 (0) |
| Stage, *n* (%) |  | |
| I | 27 (75) | 24 (77) |
| II | 4 (11) | 3 (11) |
| III | 5 (14) | 2 (6) |
| IV | 0 (0) | 2 (6) |
| Distant metastasis, *n* (%) | 0 (0) | 2 (6) |
| **SD = standard deviation** | | |

PRCC, papillary renal cell carcinoma; WHO/ISUP, World Health Organization/International Society of Urological Pathology.

**Table S3.** Immune profiling of ABCC2-high PRCCs stratified into “upper-end” and “lower-end” groups.

| **Immune cells significantly higher* in ABCC2-high PRCCs (upper end)** | **Immune cells significantly higher* in ABCC2-high PRCCs (lower end)** |
| --- | --- |
| CD4 T cells memory resting  Macrophages M0  Macrophages M2  Mast cells resting  Neutrophils | Monocytes  Macrophages M1  Eosinophils |
| **p* value < 0.05 | |
